# Soil Biogeochemical Cycle Couplings Inferred from a Function-Taxon Network

**DOI:** 10.34133/2021/7102769

**Published:** 2021-03-10

**Authors:** Bin Ma, Erinne Stirling, Yuanhui Liu, Kankan Zhao, Jizhong Zhou, Brajesh K. Singh, Caixian Tang, Randy A. Dahlgren, Jianming Xu

**Affiliations:** ^1^Institute of Soil and Water Resources and Environmental Science, College of Environmental and Resource Sciences, Zhejiang University, Hangzhou 310058, China; ^2^Zhejiang Provincial Key Laboratory of Agricultural Resources and Environment, Zhejiang University, Hangzhou 310058, China; ^3^Hangzhou Global Scientific and Technological Innovation Center, Zhejiang University, Hangzhou 310058, China; ^4^Acid Sulfate Soils Centre, School of Biological Sciences, The University of Adelaide, Adelaide, SA 5005, Australia; ^5^Institute for Environmental Genomics, Department of Microbiology and Plant Biology, and School of Civil Engineering and Environmental Sciences, University of Oklahoma, Norman, OK, USA; ^6^Global Centre for Land-Based Innovation, Hawkesbury Institute for the Environment, Western Sydney University, Penrith, NSW 2750, Australia; ^7^Department of Animal, Plant and Soil Sciences, La Trobe University, Melbourne Campus, Bundoora, VIC 3086, Australia; ^8^Department of Land, Air and Water Resources, University of California, Davis, 95616 CA, USA

## Abstract

Soil biogeochemical cycles and their interconnections play a critical role in regulating functions and services of environmental systems. However, the coupling of soil biogeochemical processes with their mediating microbes remains poorly understood. Here, we identified key microbial taxa regulating soil biogeochemical processes by exploring biomarker genes and taxa of contigs assembled from metagenomes of forest soils collected along a latitudinal transect (18° N to 48° N) in eastern China. Among environmental and soil factors, soil pH was a sensitive indicator for functional gene composition and diversity. A function-taxon bipartite network inferred from metagenomic contigs identified the microbial taxa regulating coupled biogeochemical cycles between carbon and phosphorus, nitrogen and sulfur, and nitrogen and iron. Our results provide novel evidence for the coupling of soil biogeochemical cycles, identify key regulating microbes, and demonstrate the efficacy of a new approach to investigate the processes and microbial taxa regulating soil ecosystem functions.

## 1. Introduction

Elemental fluxes in soils are largely driven by microbially catalyzed, but thermodynamically constrained, redox reactions. Soil biogeochemical cycles are the foundation of ecosystem function and affect nutrient and energy flows that regulate productivity within both terrestrial and aquatic ecosystems [1]. Given the central role of soil microbial communities in regulating global biogeochemical processes, managing soil communities provides a powerful tool to combat several increasingly important global challenges, such as feeding the world's increasing population, soil pollution, biodiversity loss, and climate change [2]. Despite the critical importance of microorganisms in regulating soil biogeochemical processes, fundamental questions concerning the linkage between specific microbial taxa and biogeochemical functions remain poorly understood, thereby limiting scientific advances. A major challenge is rooted in the fact that the vast majority of soil microbial taxa remain uncharacterized, hindering our efforts to untangle their unresolved roles in biogeochemical functions [3]. Given that different microorganisms perform a myriad of roles in biogeochemical processes, linking biogeochemical functions to specific soil microbial taxa is critical for improving management and conservation policies to maintain key ecosystem functions.

From the simplest perspective, microbial biogeochemical cycling is composed of redox half-cell reactions. The substrates for coupled half-cells in biologically driven redox reactions are sourced from the environment directly or as waste products of microbial metabolism, thereby constructing linked metabolic systems driving biogeochemical cycles [4, 5]. While biogeochemical cycles logically interact with each other, they are generally studied in isolation. Theoretical coupling among soil biogeochemical cycles can be inferred from thermodynamic modeling [6], as well as some field evidence. For example, methane oxidation is linked with N cycling [7], C utilization rates vary with P bioavailability [8], and ammonia oxidation couples with ferric reduction or thiosulfate reduction under anaerobic conditions [9, 10]. However, the coupling of some biogeochemical cycles is predicted to exist only on a thermodynamics basis [6]. Moreover, in all these approaches, there is little information concerning the specific microbes regulating the coupling of biogeochemical cycles.

Functional genes that encode enzymes for specific redox reactions have the potential to reveal the functioning of specific microbes in various biogeochemical cycles. However, the coupling of biogeochemical cycles is often spatially or temporally separated, hindering the efficiency of coupled oxidation/reduction reactions [11]. Hence, microbial taxa have the potential to greatly enhance the understanding of coupling between biogeochemical cycles when their genomes contain genes for both components of the coupled cycles. Despite pioneering conceptual research [12], there is a paucity of information concerning the connection of microbial community composition to biogeochemical functions. Recent advances in cultivation-independent metagenomic sequencing provide a powerful new approach to link biogeochemical functioning with their marine microbial drivers [13], opening opportunities to explore the coupling of soil biogeochemical processes with their specific microbial taxa.

To examine the coupling of soil biogeochemical functions and their potential microbial drivers, we associated biomarker genes for soil biogeochemical cycles with their corresponding microbial taxa by investigating the functional profiles and taxonomy of contigs assembled from the metagenomes of 45 forest soils from forests along a 4000 km latitudinal transect (18 to 48° N) in eastern China (Figure S1). This dataset contains soils spanning five Köppen climate classifications (Aw, Cfa, Cwa, Dwa, and Dwb) ranging from tropical to warm summer continental. We hypothesize that certain biogeochemical cycle couplings are driven by specific microbial taxa and that these taxa are affected by soil properties. Our results provide critical insights into the complex interconnection among biogeochemical cycles and their microbial drivers in forest soil ecosystems.

## 2. Results

### 2.1. Functional Gene Composition and Diversity

**Figure 1:**
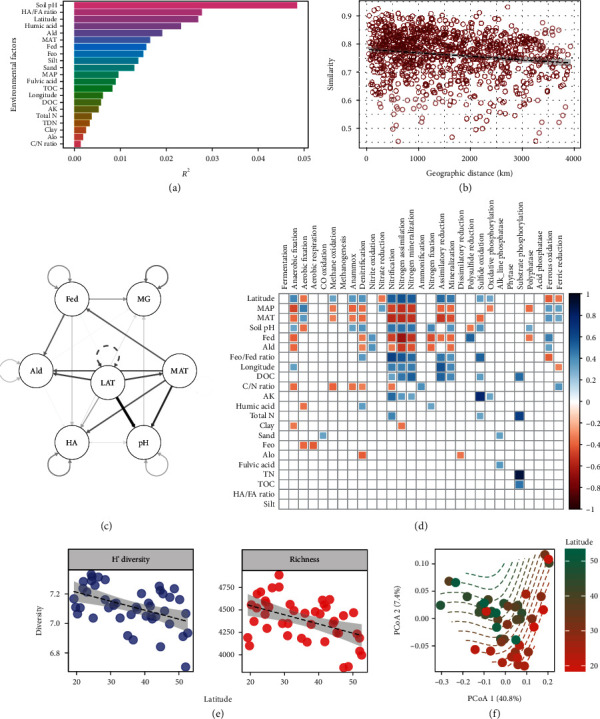
Environmental drivers of soil metagenomes. (a) The impact of environmental factors. HA: humic acid; FA: fulvic acid; Ald: dithionite extractable Al; MAT: mean annual temperature; Fed: dithionite extractable Fe; Feo: amorphous sesquioxide Fe; MAP: mean annual precipitation; TOC: total organic carbon; DOC: dissolved organic carbon; AK: available K; TDN: total dissolved N; Alo: amorphous sesquioxide Al. (b) Distance-decay of Bray-Curtis similarity of metagenomes. (c) Structure equation models (SEM) between soil metagenomes (MG) and major environmental/soil drivers, including LAT, MAT, HA, soil pH, Ald, and Fed. (d) Spearman's correlation between biomarker genes of biogeochemical process and environmental/soil factors. (e) Correlation of *H*′ diversity and richness of functional genes in forest soil metagenomes. (f) Principal coordinate analysis (PCoA) of functional genes in forest soil metagenomes; point color represents latitude of sampling sites.

Multiple regressions on the distance matrix analysis showed that the variation of soil metagenomes along the latitudinal transect was mainly determined by latitude, humic acid, soil pH, mean annual temperature, and dissolved Al and Fe (Figure 1(a)). The impact of distance on soil metagenomes was less than that of environmental variables (Figure 1(b)). Latitude significantly correlated with most functional genes for biogeochemical processes, potentially via a linkage to soil pH (Figures 1(c) and 1(d)). As the primary agent generating the environmental gradient (Figure S2), latitude correlated negatively with functional gene diversity (richness: *r* = 0.43, *P* = 0.003; *H*′ diversity: *r* = 0.42, *P* = 0.004) (Figure 1(e)); functional gene composition shifted along the transect (Figure 1(f)).

### 2.2. C, N, P, S, and Fe Biogeochemical Cycles

**Figure 2:**
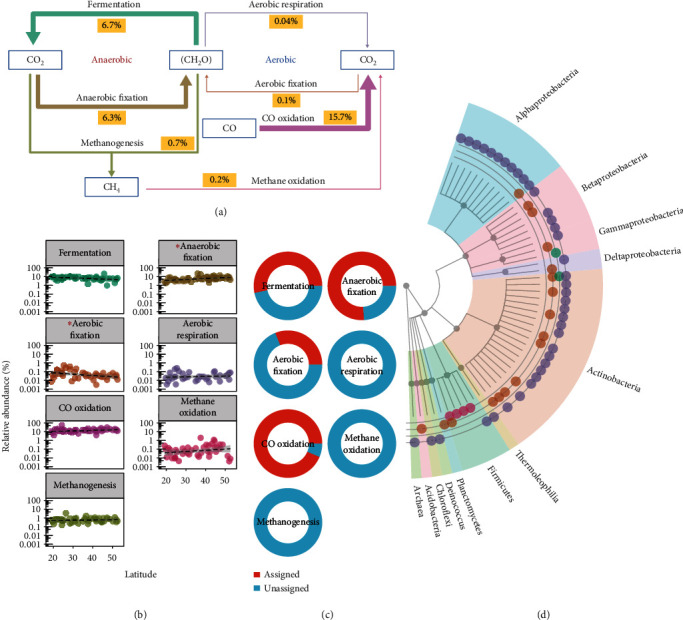
Carbon biogeochemical cycle genes in forest soils. (a) Carbon biogeochemical cycle; arrow thickness indicates proportion of biomarker gene contigs for corresponding pathways. (b) Linear relationships between contigs and latitude. Asterisks indicate significant correlation with latitude (*P* < 0.05, *n* = 45). (c) Proportion of contigs assigned to known taxa. (d) Distribution of known taxa.

Carbon-cycling biomarker genes were dominated by aerobic CO oxidation (*coxL*), anaerobic fermentation (*LDH*), and anaerobic C fixation (*KorB*) (15.7, 6.7, and 6.3% of all contigs, respectively; Figure 2(a)). Latitude correlated positively with anaerobic C fixation (*P* = 0.01) and negatively with aerobic C fixation (*PRK*) (*P* = 0.01; Figure 2(b)). Four of the seven biomarker genes had associated contigs assigned to known genera (Figure 2(c)). Of the biomarker genes with identifiable taxa (Figure 2(d)), CO oxidation was associated with the broadest range of taxa (Figure 2(d)).

**Figure 3:**
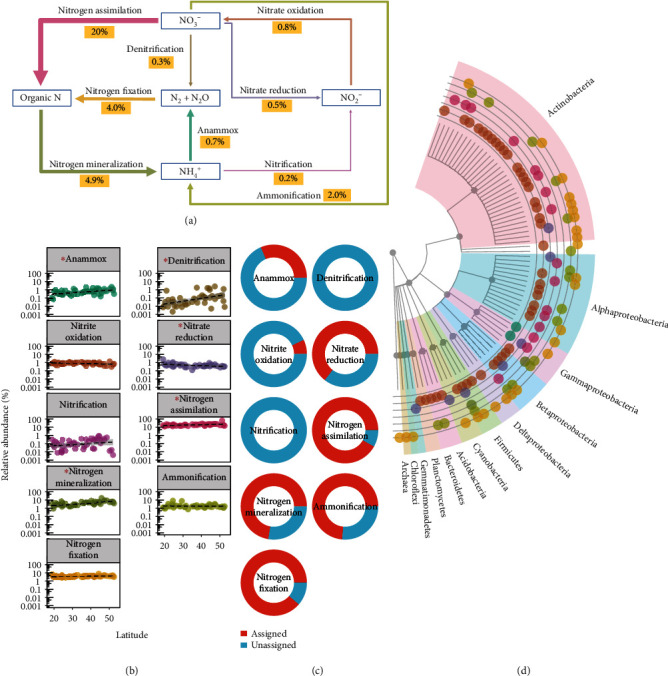
Nitrogen biogeochemical cycle genes in forest soils. (a) Nitrogen biogeochemical cycle; arrow width indicates proportion of biomarker gene contigs for corresponding pathways. (b) Linear relationships between contigs and latitude. Asterisks indicate significant correlation with latitude (*P* < 0.05, *n* = 45). (c) Proportion of contigs assigned to known taxa. (d) Distribution of known taxa.

Nitrogen-cycling biomarker genes were dominated by N assimilation (*glnA*) (20% of all contigs; Figure 3(a)). Latitude correlated positively with anammox (*P* < 0.05), N mineralization (*P* < 0.001), denitrification (*norB*) (*P* < 0.05), and N assimilation (*P* = 0.01), but negatively with nitrate reduction (*modA*) (*P* < 0.01; Figure 3(b)). Seven of the nine biomarker genes had associated contigs assigned to known genera; however, both anammox (*ccoN*) and nitrite oxidation (*narG*) had less than a third of their contigs assigned (Figure 2(c)). Of the biomarker genes with identifiable taxa (Figure 3(d)), Actinobacteria were the most frequently assigned taxa.

Phosphorus-cycling biomarker genes were dominated by substrate phosphorylation (*Ptsl*) and oxidative phosphorylation (*ppk*) (10.9 and 6.2% of all contigs, respectively; Figure S3a) but were not correlated with latitude (*P* > 0.05, Figure S3b). All biomarker genes had at least 23% of contigs assigned to known genera (Figure S3c). Actinobacteria (10 genera) and Proteobacteria (9 genera) played a dominant role in oxidative phosphorylation, whereas Firmicutes (5 genera) contributed to substrate phosphorylation (Figure S3d).

Sulfur-cycling biomarker genes were dominated by dissimilatory sulfate reduction (*Fer4*) (6.3% of all contigs; Figure S4a). Latitude correlated positively with S mineralization (*sseA*) (*P* = 0.03) and negatively with polysulfide reduction (*NrfD*) (*P* = 0.03; Figure S4b). Four of the five biomarker genes had contigs assigned to known taxa (Figure S4c). The biomarker genes with a high proportion of assigned taxa were associated with a wide variety of taxa (Figure S4d), namely, Proteobacteria (26 genera) and Actinobacteria (22 genera).

Iron-cycling biomarker genes were dominated by ferrous oxidation (*Ferritin*) (4.7% of all contigs; Figure S5a). Latitude correlated negatively with both biomarker genes for Fe cycling (*P* < 0.02; Figure S5b); the majority of taxa associated with these genes were assigned classifications with the bulk of organisms belonging to Proteobacteria (Figure S5c, d).

### 2.3. Linking Biogeochemical Processes to Microbial Taxa Using a Function-Taxa Bipartite Network

**Figure 4:**
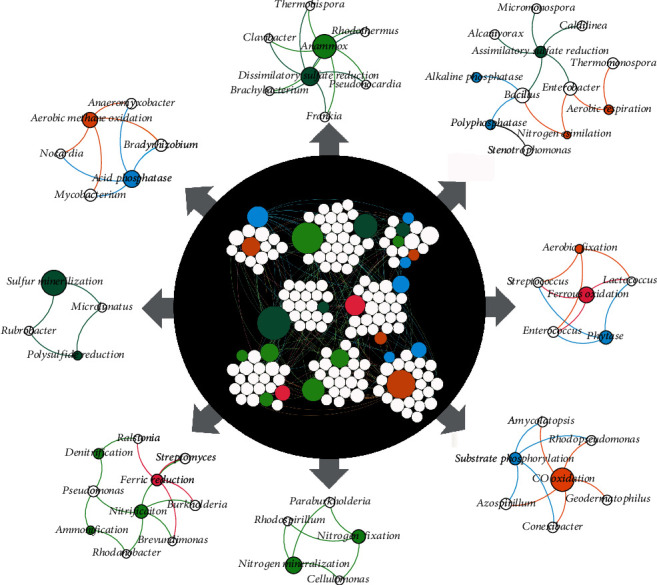
Function-taxa bipartite network for biogeochemical cycling processes. Subnetworks were induced from the functional nodes within each biogeochemical module and their coassociating taxa.

The function-taxa bipartite network between genes linked to biogeochemical processes and their associated taxa formed eight clusters (Figure 4). Biogeochemical processes are potentially driven by the taxa present within the same cluster. Therefore, taxa within a cluster are potentially regulating both biogeochemical processes and the coupling processes. Most clusters contained processes from at least two biogeochemical cycles; the maximum number in a cluster was four (C, N, P, and S). There was distinct clustering for processes requiring similar redox states.

Carbon and P cycling presented together in three clusters (Figure 4). The first cluster, between genes linked to aerobic methane oxidation and acid phosphatase cycles, was driven by Proteobacteria (*Anaeromyxobacter* and *Bradyrhizobium*) and Actinobacteria (*Mycobacterium* and *Nocardia*). The second cluster, between genes linked to aerobic C fixation and phytase, was driven by Firmicutes (*Streptococcus*, *Lactococcus*, and *Enterococcus*). The third cluster, between genes linked to CO oxidation and substrate phosphorylation, was driven by Actinobacteria (*Amycolatopsis* and *Conexibacter*) and Proteobacteria (*Azospirillum* and *Rhodopseudomonas*).

Nitrogen and S cycling occurred together in two clusters (Figure 4). The first association, between genes linked to anammox and dissimilatory sulfate reduction, was driven by Actinobacteria (*Thermobispora*, *Pseudonocardia*, *Brachybacterium*, and *Frankia*), whereas the second association, between genes linked to N assimilation and assimilatory sulfate reduction, was driven by Firmicutes (*Bacillus*). Finally, genes linked to nitrification, denitrification, and ferric reduction presented together in one cluster; this relationship was driven by Proteobacteria (*Ralstonia*, *Burkholderia*, and *Brevundimonas*).

## 3. Discussion

Our results provide novel empirical evidence that there is coupling between the biogeochemical cycles that play significant roles in regulating soil microbial functions in forest ecosystems. The results support the hypothesis that certain biogeochemical cycle couplings are associated with specific microbial taxa. Identified soil microbial functions are consistent with typical latitudinal biodiversity gradients and indicate that these gradients are regulated by environmental variables such as precipitation and soil pH [14]. Of the measured environmental variables, soil pH, a well-known predictor of species richness in soil bacterial and fungal communities [15, 16], was a significant regulator of genes associated with key biogeochemical cycles.

Soil pH is an edaphic variable sensitive to latitude due to the influence of rainfall and temperature on weathering (Figure S2) as soils in high-rainfall regions tend to have greater levels of acidity, leaching of base cations and humification of organic matter [17]. This relationship is clearly evident in eastern China where pH increases by ~5 units with latitude [18]. In addition to mediating soil community composition [19], soil pH is known to influence specific microbial-mediated biogeochemical processes, such as microbial denitrification and S mineralization. Consistent with our results, denitrification rates were shown to decrease with decreasing pH [18], whereas higher soil pH increases S mineralization rates [20]. In addition, our results indicate that polysulfide reduction was negatively correlated with latitude; polysulfide reduction is mainly driven by *Clostridium* [21], which is an acidophilic genus adapted to the low-pH region of our study. The role of pH in soil biogeochemical reactions is multifaceted. Bacteria, for example, are often constrained to relatively tight optimal pH ranges [19], and the expression of certain genes is similarly constrained to specific pH ranges [22]. Soil pH affects the strategies that microbes can use to acquire nutrients and extremes of pH require microbes to spend additional energy and resources maintaining their cell's physicochemical integrity [23].

Notably, a prevalence of anoxic microenvironments was inferred by the high proportion of CO oxidation, fermentation, and anaerobic fixation genes across all sampling sites even though all soils were considered well drained and oxygenated. It is possible that oxic degradation of soil organic matter under conditions of low gas permeability (e.g., sites within soil structural units or surrounding recently dead roots) caused a prevalence of transient anoxic microniches (hotspots/hot moments) [24]. Although CO is toxic to many organisms, numerous cultured and molecular ecological approaches have revealed an unexpectedly diverse group of soil bacteria that have the ability to use CO as an energy source, including members of phyla Proteobacteria and Actinobacteria [25]. The prevalent role of facultative anaerobes is supported by the similar proportions of oxidative and substrate phosphorylation genes, indicating both anaerobic and aerobic generation of energy [26]. An abundance of dissimilatory S reduction genes also suggests a preponderance of anoxic soil conditions as dissimilatory sulfate reactions are typically facilitated by sulfate-reducing microbes in anaerobic environments [27].

Nitrogen cycling is well known to be affected by edaphic properties; N mineralization, anammox, nitrification, and denitrification marker genes were all significantly correlated with latitude in our study. Although N mineralization has previously been negatively associated with latitude [28], we found that *GDH2* increased with latitude. The N assimilation marker gene similarly increased with latitude. Increased relative abundance of *GDH2* may be associated with lower-quality organic matter at high latitudes as glutamate dehydrogenase activity has been shown to decrease as the C : N ratio increases [29, 30]; increased activation energy requirements in colder climates may also lead to an increase in gene expression [31]. Marker genes associated with anammox similarly increased with latitude; however this may have been in response to increasing pH and associated Fe limitation [32]. The denitrification marker gene also increased with latitude; however, this is in contrast with established literature in which denitrification increases with temperature and therefore tends to decrease with latitude [33, 34].

This study provides novel evidence that biogeochemical processes are coupled via associations among individual taxa possessing multiple functional genes, as observed from the function-taxa bipartite network. The most frequent potential association between biogeochemical cycles was between P and C. Methane oxidation was previously shown to correlate positively with P concentration in soil/sediment [7] and acid-phosphatase genes have been shown to be present in all methanotrophic bacteria [7]. One of the predicted driver taxa, *Bradyrhizobium*, was reported to enhance acid phosphatase activity in arbuscular mycorrhizal fungi [35] and has a known association with methanotrophic bacteria in rice paddy soils [36]. Similarly, a positive response was previously observed between P concentration and C fixation in seawater [31]. Another predicted driver, *Streptococcus*, is associated with phytase production and mineralization of phosphate [37]. Among the predicted regulating genera, only *Rhodopseudomonas* appears to be associated with CO and CO_2_ metabolism [38].

Prominent coupling also appears to occur between N and S cycling and between N and Fe cycling. Of the relationships between these biogeochemical cycles, sulfate reduction is known to couple with anammox when removing N from wastewater. The predicted microbial drivers *Thermobispora*, *Pseudonocardia*, *Brachybacterium*, and *Frankia* have been implicated in anammox reactions [39], but none of these bacteria appear to be involved in S cycling. Curiously, *Ralstonia* has been shown to use thiosulfate during anaerobic ammonia oxidation but was directly associated with ferric reduction and denitrification in our cooccurrence network [10]. However, it is possible for one cycle to affect another cycle simply via its limited availability in soil, as with the association between assimilatory sulfate reduction and P nutrition, whereby S deficiency increases the activity of polyphosphatase in some microorganisms [40]. Of the drivers predicted to affect coupling between genes involved in N- and S-cycling processes, *Bacillus* was associated with the highest number of pathways. *Bacillus* affects the growth of other microbes in culture through assimilatory sulfate reduction processes [41]. Other genera in the cluster containing *Bacillus* and *Enterobacter* are implicated in dissimilatory S reduction and have the ability to efficiently reduce nitrate to ammonium [42]. Coupling between ferric reduction and ammonium oxidation is reported in both paddy [43] and upland [44] soils, but our predicted microbial drivers have not been reported to drive ferric reduction process.

This study demonstrates that the potential coupling of biogeochemical processes with microbial taxa capable of generating functional genes facilitates multielement transformations within a wide range of forest soils. However, the coupling of biogeochemical processes among different taxa, which is often spatially or temporally separated, cannot be discovered with this approach. Moreover, unraveling coupled interactions between biogeochemical processes and the mitigating microbial taxa sheds light on elucidating the importance and role of the unculturable microbial ‘dark matter.' Furthermore, a significant limitation of this work is that the metagenomic predicted potential couplings of biogeochemical processes are not experimentally validated due to a large proportion of microbial ‘dark matter.' In addition, while using marker genes to assess the rate-limiting step of each process gives us an estimation of community capacity to process biogeochemical reactions, it is possible that an analysis of alternative marker genes would show an alternative response to edaphic gradients.

While the use of undisturbed sites from a single land use type provides a more constrained approach for deciphering the effects of edaphic gradients on soil microbial processes (as opposed to human-induced effects), we cannot know if these relationships hold true for other land uses. In addition to increasing the variety of landscapes sampled, future focus could employ element probes, such as stable isotope probes, for validating coupling of biogeochemical processes through labeling active microbial taxa for different pathways. Additional work could also involve investigation of soil metagenomes from other ecosystems for the comparison of driving taxa between ecosystems. Overall, this study highlights a potential avenue to enhance simulation modeling of soil biogeochemical processes to inform controls of various soil functions and potential management of key taxa to achieve specific soil functions.

## 4. Materials and Methods

### 4.1. Experimental Design

To explore potential biogeochemical couplings from surveying soil metagenomes, we collected soil samples from 45 sampling sites in eastern China covering a latitude of 18° 48′ N to 48° 36′ N (Figure S1, Table S1). To minimize the anthropological influence on soil microbial functions, all sites were located within natural forest reserves. Topsoil samples (0-15 cm; the dominant rooting zone) were collected from 100 m × 100 m plots. From each plot, we collected three analytical sample replicates, each a composite of five soil cores. The methods for measuring edaphic variables have been described previously [45]. In brief, soil pH, soil texture, organic carbon, and available potassium were determined according to the protocols outlined by the Agricultural Chemistry Committee of China. Total nitrogen was determined using a Flash 2000 NC Analyzer (Thermo Scientific, MA, USA). Sesquioxides (Ald and Fed) were extracted in the dark with dithionite-citrate solution buffered with NH_4_-oxalate (pH 3.0; amorphous sesquioxides) or NaHCO_3_ (amorphous sesquioxides–Alo and Feo) and measured using atomic absorption spectrometry (ContrAA 700, Jena, Germany). The data for mean annual precipitation and temperature were sourced from WorldClim (http://worldclim.org).

### 4.2. Metagenomic Dataset

Details of the metagenomic data acquisition have been previously described [18]. In brief, shotgun sequencing of metagenomic DNA produced a total of ~1.5 billion paired-end reads (read length = 150 bp). The raw shotgun sequencing reads were preprocessed using ngsShoRT v2.1 [46], and whole-genome *de novo* assemblies for each sample were performed using IDBA-UD; open read frame prediction and annotation were performed using Prodigal v2.50 [47]. The resulting protein translations were assigned by comparisons to Pfam 31.0 using HMMER 3 [48] and KEGG release 84.0 using GhostKOALA [49]. The number of contigs greater than 500 bp in length was 253,807; they had an N50 of 1409 and a maximum length of 160699; mean coverage was 12.9 and ranged from 4.7 to 27.4 for individual samples.

### 4.3. Biogeochemical Functional Gene Analysis

Biogeochemical functional gene analyses focused on C, N, P, S, and Fe cycling processes. The genetic potential for C, N, and S cycling in the soil microbial community was analyzed using biomarker genes as reported [50] with modifications as follows. If the biomarker genes reported were not found in all 45 metagenomes annotated using the KEGG database, these biomarker genes were replaced with equivalent genes annotated using the Pfam database. The following maker genes were replaced in this manner: methanogenesis biomarker gene K14084 with PF06253, N_2_ fixation biomarker gene K02588 with PF00142, ammonification biomarker gene K05904 with PF01077, S oxidation biomarker gene K17227 with PF08770, dissimilatory sulfate reduction biomarker gene K00394 with PF13187, and polysulfide reduction biomarker gene K08352 with PF14589.

Marker genes were also selected for P and Fe biogeochemical cycles. We used PF03767 as the biomarker gene for acid phosphatase, K01077 for alkaline phosphatase, K01083 for phytase, K00937 for oxidative phosphorylation, K08483 for substrate phosphorylation, and PF00719 for polyphosphatase. For the Fe biogeochemical cycle, we used PF00210 and PF01794 as biomarker genes for ferrous oxidation and ferric reduction as catalyzed by microorganisms, respectively. Biomarker genes used in this study are compiled in Table S2. The proportions of contigs with their corresponding marker genes for each biogeochemical pathway are shown in pathway maps (Figures 2 and 3, S2-S4 inclusive).

### 4.4. Biomarker Gene Taxonomic Profiles

Contig taxa were assigned using CLARK [51]. Biomarker gene taxonomic profiles were generated from the corresponding biomarker gene contig taxa. Unassigned taxa indicate that the contigs could not be assigned to a known taxon using CLARK. Phylogenetic trees for taxa involving a biogeochemical cycle were generated with hieratical phylogenetic relationships from kingdom to order.

### 4.5. Function-Taxon Bipartite Network

The function-taxon bipartite network was constructed from the function-taxon relationships as assigned above. The network modules were clustered using the modularity calculation in Gephi with *r* = 1 [52]. Module subnetworks were induced between functional nodes and their connecting taxa.

### 4.6. Statistical Analysis

All statistical analyses were carried out using R version 3.5.0 [53]. The impact of environmental factors on functional gene composition was estimated by multiple regression on distance matrices by ecodist::MRM in R [54]. Functional gene diversity was assessed using the Shannon-Weiner ‘*H*' diversity index; gene richness was measured using the number of genes found in corresponding samples; trend significance was established by fitting a generalized linear model with stats::glm in R. Functional composition dissimilarity was analyzed using Bray-Curtis dissimilarity and visualized using principle coordinate analysis (PCoA) with the ‘vegan' package in R [55]. The effect of latitude on relative gene abundance was established using stats::glm, as above, for each biogeochemical cycle.

## Data Availability

The sequencing datasets from this study are available in the Public National Center for Biotechnology Information (NCBI) database under BioProject accession number PRJNA293484.

## Supplementary Materials

Supplementary Materials Figure S1 Location of sampling sites. Figure S2: metadata for sampling sites along the latitudinal transect. Figure S3: genes associated with phosphorus biogeochemical cycling in forest soils. Figure S4: genes associated with sulfur biogeochemical cycling in forest soils. Figure S5: genes associated with iron biogeochemical cycling in forest soils. Table S1: GPS coordinates for all sampling sites Table S2: biomarker genes of biogeochemical processes.
