# Supplementary material for: Soil Biogeochemical Cycle Couplings Inferred from a Function-Taxon Network
Source: Research (Wash D C). 2021 Mar 10;2021:7102769. doi: 10.34133/2021/7102769 (PMC7978035; doi:10.34133/2021/7102769)
Supplement: Supplementary Materials — Figure S1 Location of sampling sites. Figure S2: metadata for sampling sites along the latitudinal transect. Figure S3: genes associated with phosphorus biogeochemical cycling in forest soils. Figure S4: genes associated with sulfur biogeochemical cycling in forest soils. Figure S5: genes associated with iron biogeochemical cycling in forest soils. Table S1: GPS coordinates for all sampling sites Table S2: biomarker genes of biogeochemical processes. [file 7102769.f1.docx]

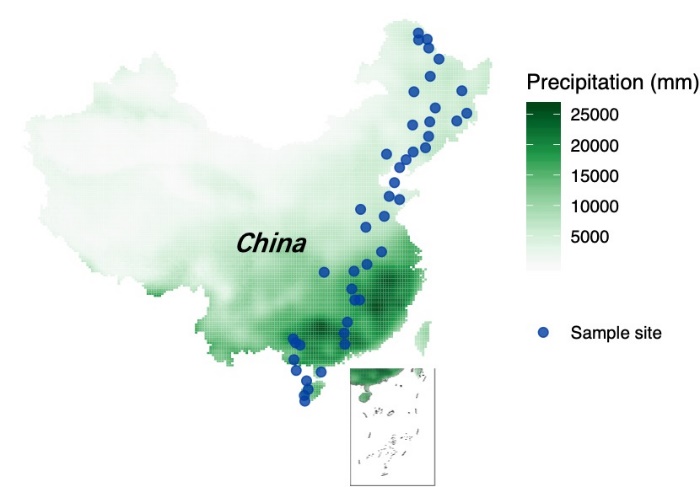


**Figure S1. Location of 45 sampling sites.**


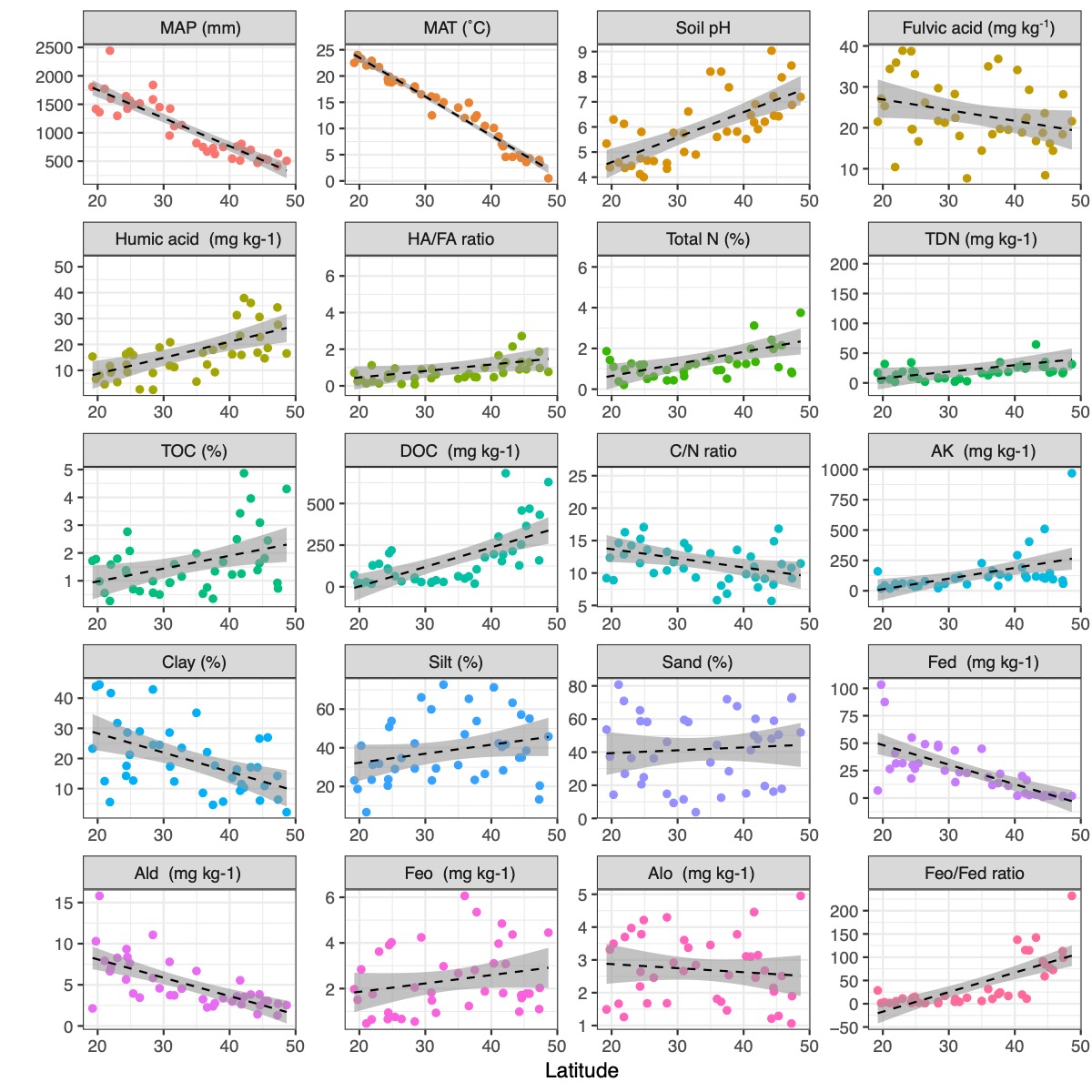


**Figure S2. Metadata for sampling sites along the latitudinal transect.** HA-humic acid; FA-fulvic acid; Ald-dithionite extractable Al; MAT-mean annual temperature; Fed-dithionite extractable Fe; Feo-amorphous sesquioxides Fe; MAP-mean annual precipitation; TOC-total organic carbon; DOC-dissolved organic carbon; AK-available K; TDN-total dissolved N; Alo- amorphous sesquioxides Al.

**
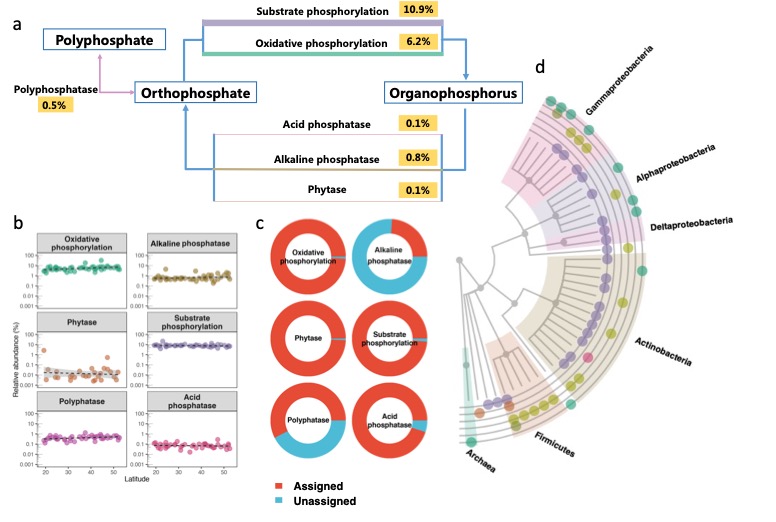
**

**Figure S3. Genes associated with phosphorus biogeochemical cycling in forest soils**. (a) Phosphorus biogeochemical cycle pathways; arrow width indicates proportion of biomarker gene contigs for corresponding pathways. (b) Linear relationships between contigs and latitude. (c) Proportion of contigs assigned to known taxa. (d) Distribution of known taxa.


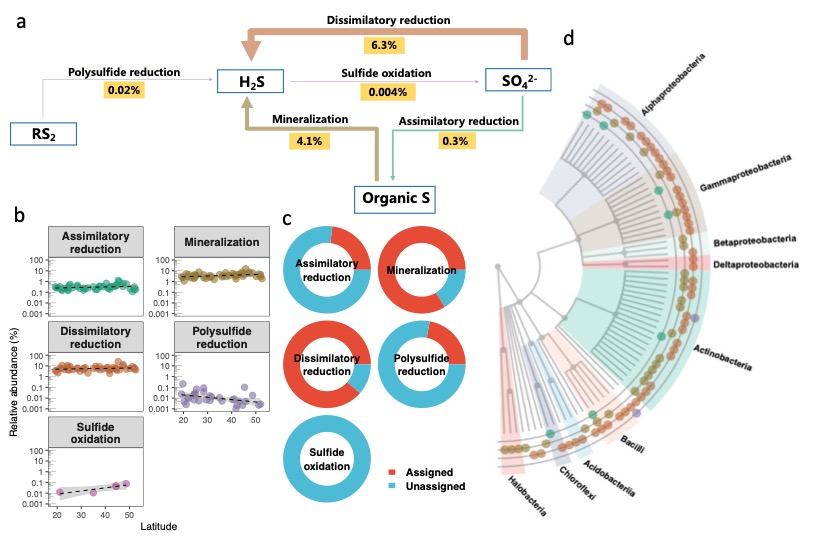


**Figure S4. Genes associated with sulfur biogeochemical cycling in forest soils.** (a) Sulfur biogeochemical cycle pathways; arrow width indicates proportion of biomarker gene contigs for corresponding pathways. (b) Linear relationships between contigs and latitude. (c) Proportion of contigs assigned to known taxa. (d) Distribution of known taxa.


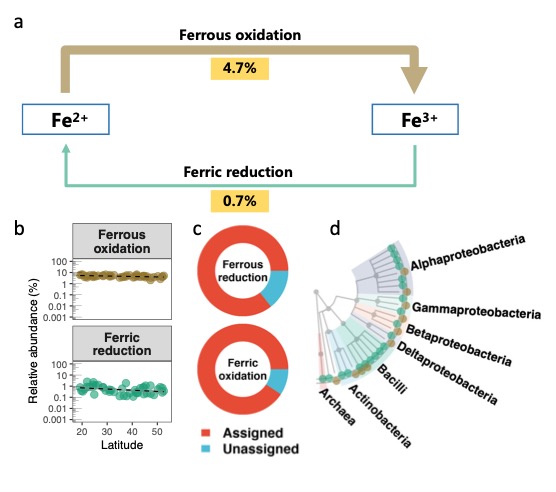


**Figure S5. Genes associated with iron biogeochemical cycling in forest soils.** (a) Iron biogeochemical cycle pathways; arrow width indicates proportion of biomarker gene contigs for corresponding pathways. (b) Linear relationships between contigs and latitude. (c) Proportion of contigs assigned to known taxa. (d) Distribution of known taxa.

**Table S1. GPS coordinates for all sampling sites**

| Sample | Longitude | Latitude | Sample | Longitude | Latitude |
| --- | --- | --- | --- | --- | --- |
| V1 | 126.1247 | 48.6509 | V24 | 112.2898 | 30.9050 |
| V2 | 130.2527 | 47.3398 | V25 | 115.8927 | 29.3888 |
| V3 | 130.8958 | 45.3086 | V26 | 116.9176 | 28.3973 |
| V4 | 124.0023 | 47.2539 | V27 | 116.2927 | 28.3876 |
| V5 | 126.7833 | 45.7893 | V28 | 115.3204 | 26.3879 |
| V6 | 129.5898 | 44.6060 | V29 | 114.9126 | 25.3985 |
| V7 | 126.0660 | 44.5282 | V30 | 114.9936 | 24.3852 |
| V8 | 123.8310 | 44.2441 | V31 | 108.2600 | 24.8700 |
| V9 | 125.5090 | 42.1828 | V32 | 108.5600 | 24.5200 |
| V10 | 125.9139 | 43.2116 | V33 | 109.1500 | 24.3100 |
| V11 | 123.9000 | 41.8000 | V34 | 108.3500 | 22.9900 |
| V12 | 120.4300 | 41.6000 | V35 | 108.6500 | 22.0100 |
| V13 | 122.9800 | 41.1000 | V36 | 111.9000 | 21.8667 |
| V14 | 122.1300 | 40.3900 | V37 | 110.0000 | 21.0667 |
| V15 | 121.4600 | 39.0200 | V38 | 110.2000 | 20.2833 |
| V16 | 120.7663 | 37.7789 | V39 | 109.7064 | 19.7429 |
| V17 | 122.1203 | 37.4890 | V40 | 109.7799 | 19.2315 |
| V18 | 117.0470 | 36.6002 | V41 | 127.2660 | 50.2129 |
| V19 | 117.7290 | 34.9834 | V42 | 125.9296 | 51.2123 |
| V20 | 120.1311 | 35.9731 | V43 | 125.7328 | 52.0126 |
| V21 | 119.7810 | 32.7556 | V44 | 124.5823 | 52.5719 |
| V22 | 117.8717 | 31.6206 | V45 | 124.6095 | 51.9504 |
| V23 | 116.1897 | 30.9966 |  |  |  |

**Table S2. Biomarker genes of biogeochemical processes.**

| Element | Pathways | Biomarker | Full name | Short name |
| --- | --- | --- | --- | --- |
| **Carbon** | Fermentation | K00016 | L-lactate dehydrogenase | *LDH* |
|  | Anaerobic C fixation | K00175 | 2-oxoglutarate/2-oxoacid ferredoxin oxidoreductase subunit beta | *KorB* |
|  | Aerobic C fixation | K00855 | Phosphoribulokinase | *PRK* |
|  | Aerobic respiration | K02256 | Cytochrome c oxidase subunit 1 | *COX1* |
|  | CO oxidation | K03520 | Aerobic carbon-monoxide dehydrogenase large subunit | *coxL* |
|  | Aerobic methane oxidation | K10944 | Methane/ammonia monooxygenase subunit A | *pmoA* |
|  | Methanogenesis | PF06253 | Trimethylamine methyltransferase | *MTTB* |
| **Nitrogen** | Nitrite oxidation | K00370 | Nitrate reductase / nitrite oxidoreductase, alpha subunit | *narG* |
|  | Anammox | K00404 | Cytochrome c oxidase cbb3-type subunit I | *ccoN* |
|  | Nitrogen assimilation | K01915 | Glutamine synthetase | *glnA* |
|  | Nitrate reduction | K02020 | Molybdate transport system substrate-binding protein | *modA* |
|  | Denitrification | K04561 | Nitric oxide reductase subunit B | *norB* |
|  | Nitrification | K10946 | Methane/ammonia monooxygenase subunit C | *amoC* |
|  | Nitrogen mineralization | K15371 | Glutamate dehydrogenase | *GDH2* |
|  | Nitrogen fixation | PF00142 | 4Fe-4S iron sulfur cluster binding proteins | *nifH* |
|  | Ammonification | PF01077 | Nitrite and sulfite reductase 4Fe-4S domain | *NIR* |
| **Phosphorus** | Oxidative phosphorylation | K00937 | Polyphosphate kinase | *ppk* |
|  | Alkaline phosphatase | K01077 | Alkaline phosphatase | *phoA* |
|  | Phytase | K01083 | Phytase | *phytase* |
|  | Substrate phosphorylation | K08483 | PTS system enzyme I | *Ptsl* |
|  | Polyphatase | PF00719 | Pyrophosphatase | *Pyrophosph-atase* |
|  | Acid phosphatase | PF03767 | Acid phosphatases | *acid_phosphat* |
| **Sulfur** | Assimilatory sulfate reduction | K00860 | Adenylylsulfate kinase | *cysC* |
|  | Sulfur mineralization | K01011 | 3-mercaptopyruvate sulfurtransferase | *sseA* |
|  | Sulfur oxidation | PF08770 | Sulphur oxidation protein | *soxZ* |
|  | Dissimilatory sulfate reduction | PF13187 | Ferredoxins | *Fer4* |
|  | Polysulfide reduction | PF14589 | Glycoside hydrolase | *NrfD* |
| **Iron** | Ferrous oxidation | PF00210 | Ferritin | *Ferritin* |
|  | Ferric reduction | PF01794 | Ferric reductase transmembrane component | *Ferric_ reduct* |
